# The effect of exercise-based interventions on health-related quality of life and physical function in older patients with cancer receiving medical antineoplastic treatments: a systematic review

**DOI:** 10.1186/s11556-020-00250-w

**Published:** 2020-10-19

**Authors:** Marta Kramer Mikkelsen, Carsten Bogh Juhl, Cecilia Margareta Lund, Mary Jarden, Anders Vinther, Dorte Lisbet Nielsen

**Affiliations:** 1 Department of Oncology and Hematology, Copenhagen University Hospital, Rigshospitalet, Blegdamsvej 9, 2100 Copenhagen Ø, Denmark; 2 Department of Oncology, Copenhagen University Hospital, Herlev and Gentofte Hospital, Borgmester Ib Juuls Vej 1, 2730 Herlev, Denmark; 3 Department of Physiotherapy and Occupational Therapy, Copenhagen University Hospital, Herlev and Gentofte Hospital, Borgmester Ib Juuls Vej 1, 2730 Herlev, Denmark; 4 Department of Sports Science and Clinical Biomechanics, University of Southern Denmark, Campusvej 55, 5230 Odense M, Denmark; 5 Department of Medicine, Copenhagen University Hospital, Herlev and Gentofte Hospital, Borgmester Ib Juuls Vej 1, 2730 Herlev, Denmark; 6 QD-Research Unit, Copenhagen University Hospital, Herlev and Gentofte Hospital, Borgmester Ib Juuls Vej 1, 2730 Herlev, Denmark

**Keywords:** Antineoplastic treatment, Cancer, Exercise, Older, Physical activity, Systematic review

## Abstract

Older patients with cancer are underrepresented in trials investigating the effect of exercise therapy. The aim of this systematic review was to investigate the effect of exercise therapy during medical antineoplastic treatment in older patients (≥ 65 years) with cancer. A systematic review following the Cochrane guidelines was performed. Randomized controlled trials were identified through a systematic literature search in MEDLINE, EMBASE, CENTRAL, and CINAHL up to December 2019. Study selection was performed independently by two reviewers. Four randomized controlled trials published between 2014 and 2019 were included comprising a total of 412 participants. Most participants were diagnosed with breast, prostate or colorectal cancer. The studies were characterized by large differences in design, interventions and outcomes, which prevented meta-analyses. The interventions ranged from 4 weeks to 12 months and involved both supervised and unsupervised exercise programs. Some evidence of beneficial effects from the interventions were documented on physical function, muscle strength, physical activity and cognitive function. No evidence of effects was found for health-related quality of life, aerobic capacity, body composition, cancer-related symptoms and side effects, or for any clinical outcomes. No adverse events were reported. Exercise therapy seems to be safe and feasible in older patients with cancer. However, due to a limited number of studies, small sample sizes and heterogeneity across study design, the effects of exercise in older patients with cancer receiving medical antineoplastic treatment are inconclusive.

## Introduction

The role of physical activity (PA) and exercise in cancer prevention, treatment and rehabilitation has been widely investigated and discussed in the last decades. There is increasing evidence that a physically active lifestyle lowers the risk of some cancers [1]. The strongest evidence for a risk reduction has been demonstrated in breast, endometrial, colon, gastric, kidney, bladder and esophageal cancer [1–8]. In a recently published systematic review, the relative risk reductions ranged from approximately 10 to 20% [1]. In addition to PA playing a preventive role, studies have shown that patients with higher PA levels had lower risk of breast and colon cancer-specific mortality [9–11]. After a cancer diagnosis, several studies have demonstrated that exercise can maintain or increase muscle mass and strength, aerobic capacity, functional mobility and health-related quality of life (HRQoL) in patients with cancer and cancer survivors [12, 13]. However, most of the conducted studies have been performed among younger patients with cancer [14–16]. In 2016, the Cancer and Aging Research Group provided a review focusing on exercise-based trials among older adults with cancer [16]. Only seven studies were identified in which the mean age of the study populations reached 60 years or more. Furthermore, most studies included cancer survivors in the post-treatment period [16]. Similarly, in a recently published systematic review focusing on effects of nutrition and physical activity interventions among older patients with cancer, the researchers had to lower their initial age cut-off criterion from ≥70 to ≥60 years of age, as they did not find any studies in the initial search. Even with a cut-off of ≥60 years and with inclusion of all treatments (and non-treatment) only a small number of studies were identified [17].

The risk of cancer increases with aging [18]. In 2012, 47.5% of all cancers worldwide were diagnosed among older adults (≥ 65 years), while only 8% of the world population is in this age group [18]. Natural aging causes physiological changes, including decreased organ function [19–21], loss of muscle mass [22] and reduced bone mineral density [23]. In addition, comorbidity increases with age and heightens the risk of disability and mortality [24]. Age-related changes in health place older adults in additional risk of short and long-term disability when diagnosed with cancer [25]. Due to recruitment challenges and exclusion criteria, older patients with cancer have been underrepresented in exercise-based intervention studies [16]. Therefore, it is unlikely that these trial results can be generalized to the older cancer population. The primary aim of this systematic review was to investigate the effect of exercise-based interventions on HRQoL and physical function. Secondarily, we looked at the effects of exercise interventions on aerobic capacity, muscle strength, body composition, cancer and treatment-related side effects, feasibility of the interventions, and survival/mortality in older patients (≥ 65 years) with cancer during medical antineoplastic treatment.

## Methods

### Registrations

The study protocol was registered at the international prospective register of systematic reviews PROSPERO on April 11, 2019 (Reg. ID: CRD42019128349).

### Data sources and searches

A comprehensive search was conducted in four databases: MEDLINE, EMBASE, CENTRAL and CINAHL. A matrix that consisted of the following search focus was conducted and used in all the databases: a) exercise, b) cancer, c) older adults, and d) randomized controlled trials (RCTs). Applied search words according to the search focus are shown in Additional file 1: Appendix A. A broad search strategy was chosen to capture all relevant exercise-based studies, as we expected only few eligible studies in this research field based on the findings from Kilari et al. [16] The search was conducted using free text words and indexed terms for search focus a-c. RCTs were identified using the Cochrane Collaboration highly sensitive search strategy. In addition, a manual search of references in the included studies was conducted. No restrictions were used for either language or time of publication. The search was updated on December 9, 2019.

### Study selection criteria

Studies were included if they fulfilled the following criteria: 1) Investigated the effect of an exercise-based intervention defined as resistance training, aerobic, balance or flexibility exercises. Programs including active stretching exercises were accepted (e.g. focusing on specific cancer-related joint problems/decreased range of motion), whereas programs solely including passive stretching exercises were excluded. Mixed exercise interventions consisting of a specific exercise program such as pilates or yoga were accepted. Multimodal programs were accepted if at least 50% of the intervention time included exercise; 2) Included patients with cancer in medical antineoplastic treatment. All types of solid cancers and hematologic malignancies were included. Medical antineoplastic treatment was defined as chemotherapy, endocrine therapy, targeted therapy, immunotherapy or combinations of these. Studies that included different treatment regimens were accepted if at least 50% of the participants received medical antineoplastic treatments; 3) Exclusively included older adults (≥ 65 years); 4) Were RCTs, including randomized cross-over and feasibility trials; 5) Reported on at least one of the following outcomes; HRQoL, physical function, aerobic capacity, muscle strength, body composition, cancer and treatment-related symptoms and side effects, or survival/mortality.

### Outcomes

The primary outcomes of interest were changes in HRQoL and in physical function. Measures of HRQoL included both generic and cancer specific patient-reported outcome measures (PROMs). Physical function is closely related to activities of daily living, and all measures of physical functioning (including PROMs) were included. Secondary outcomes included changes in aerobic capacity (maximal oxygen uptake (VO2max) or substitute measures), muscle strength, body composition, cancer and treatment-related symptoms and side effects, feasibility (recruitment rate, retention, drop-outs and adherence), safety, and survival/mortality. Additional outcomes that were investigated in the included studies will also be reported.

### Literature searches and study selection

Two authors [MKM and CBJ] conducted the literature searches in the chosen databases. Two authors [MKM and DLN] independently reviewed all identified studies from the literature searches. In the first phase, titles and abstracts were reviewed. After a consensus meeting, studies that were identified as relevant were reviewed in full text and included if they fulfilled the criteria. Any disagreements were resolved by discussion between three authors [MKM, CBJ and DLN].

### Risk of bias

Risk of bias in the included studies was assessed using the Cochrane Risk of Bias Tool (RoB2) [26]. RoB2 is the latest updated version of the Cochrane risk-of-bias tool for randomized trials. It is used to assess the risk of bias due to the randomization process, deviations from the intended interventions, missing outcome data, measurement of the outcome, and selection of reported results. The assessment is conducted using a series of signaling questions and results in judgements of “low”, “some concerns” or “high” risk of bias [26]. The overall risk of bias was judged as “high risk” if one or more domains were judged “high risk” of bias, or if three or more domains were judged as “some concerns”. Low risk of bias was only judged in studies that were judged “low risk” of bias in all five domains. Risk of bias assessment for each study was performed independently by two authors [MKM and DLN]. Any disagreements were resolved by discussion, or if necessary, by consulting a third author [CBJ].

### GRADE assessment

We intended to perform meta-analyses and to assess the overall quality of evidence using the Grading of Recommendation, Assessment, Development and Evaluation tool (GRADE) [27], as described in the preregistration of the protocol. However, due to the substantial differences between included studies regarding participants, interventions and outcomes, an overall evaluation of the effect of exercise interventions was not performed.

### Statistics

As the included studies were characterized by large differences in study design, interventions and outcomes, meta-analysis was not performed and instead a narrative synthesis approach was used. The results are presented as means with standard error (SE) or medians with interquartile range (IQR).

## Results

**Fig. 1:**
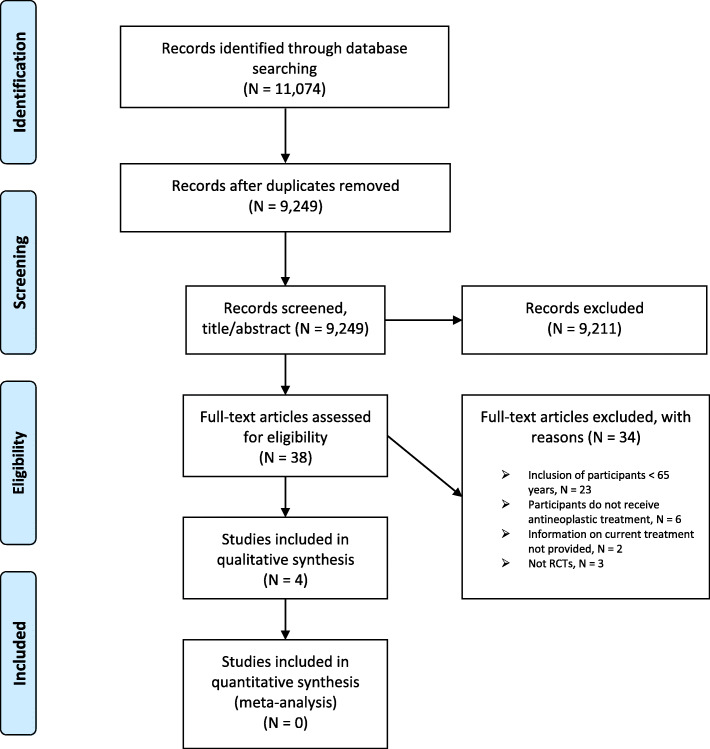
PRISMA Flow Diagram. Flowchart showing the identification of studies from the systematic literature searches

The initial search in MEDLINE, EMBASE, CENTRAL and CINAHL identified 11,074 studies. After removing duplicates, 9249 studies remained. Titles and abstracts were screened in the first review phase, leaving 38 studies for further reviewing. In full text review, a total of 34 studies were excluded, including two otherwise eligible studies in which no information on current treatment was provided [28, 29]. Finally, only four studies were deemed eligible for inclusion. A ‘Preferred Reporting Items for Systematic Reviews and Meta-Analysis’ (PRISMA) flowchart of the identification process is shown in Fig. 1.

### Study characteristics

The design and setup of the included studies are summarized in Table 1. The studies were conducted in France [30], Canada [31], USA [32], and Japan [33], and published from 2014 to 2019. The studies were all RCTs, however, one study was a pilot RCT [32]. One study [31] recruited patients who were a part of a single blinded pilot study called CANcer EXercise (CANEX) that investigated the impact of a mixed exercise program (unpublished study), with no further explanation provided. Two studies included patients with various cancers [30, 31], one study included patients with breast or prostate cancer [33], and one study solely included patients with prostate cancer [32]. In three studies the inclusion criterion was age of ≥65 years [31–33], while one included individuals ≥70 years [30]. Two studies excluded patients who were already physically active [31, 32], and two excluded patients with an Eastern Cooperative Oncology Group (ECOG) performance status (PS) > 1 [33] and > 2 [30], respectively.

**Table 1:** Study designs

Author, year, country	Diagnoses	Age, years	Exclusion criteria	Intervention				Control group	Timing of tests	Outcomes
				Delivery	Type	Frequency & duration	Intensity			
**Arrieta** et al [30], 2019 France	Various cancers	≥ 70	ECOG PS > 2 Serious psychiatric or cognitive problems Inability to walk Palliative care	Home-based, unsupervised	Individualized phoned PA advice (on resistance and aerobic exercises) and booklet with exercises	Telephone advice: 2X a month for 6 months, and then 1X a month Exercise: starting at 2X a week → hereafter adjusted to each participant Duration: 12 months	Adjusted to each participant’s capability	Usual care	Baseline 3 months 6 months 12 months 18 months 24 months	Physical function^a^ PA^b^ Cognition Falls Hospitalizations Institutionalizations Mortality
**Maréchal** et al [31], 2019 Canada	Various cancer	≥ 65	Physically active (≥ 75 min. Per week) Surgery planned within 6 months Uncontrolled hypertension Use of beta-blockers	Supervised and unsupervised	Aerobic exercises and resistance training	3X a week for 12 weeks Time: Aerobic: 40 min. RT: NR	4-week progression period: aerobic workout at 70–75% of HRR → maintain RT: 2–3 sets of 10–15 rep. with 50–65% of maximal exercise load	Active static stretching	Baseline 12 weeks	Physical function Strength Aerobic fitness PA^b^ Balance Flexibility SBT^b^ PC NR^a^
**Sajid** et al [32], 2016 USA	Prostate cancer	≥ 65	Cognitive impairment Already performing or planning to perform exercise > ×1 weekly	Home-based, unsupervised	EXCAP: walking and resistance training Wii: exercise with Wii-fit	At least 5X a week for 6 weeks Time: NR	EXCAP: Walking: 60–70% of HRR and 3–5 rating of perceived exertion according to ACSM scale. Instructed to increase daily steps with 5–20% per week and to reach 10,000 steps per day. RT: low/moderate intensity, instructed to increase to max. 4 sets with 15 rep. Wii: same intensity as EXCAP	Usual care	Baseline 6 weeks 12 weeks	Physical function^a^ Strength Aerobic fitness PA BC
**Miki** et al [33], 2014 Japan	Breast and prostate cancer	≥ 65	ECOG PS > 1 Bone metastases Received whole brain irradiation Severe cardio-respiratory disease Central nervous system paralysis	Supervised	Speed-feedback therapy on bicycle ergometers^c^	1X a week for 4 weeks Time: 5 min.	Exercise load: 20 watts with maximum rotations of 80 rpm	Usual care	Baseline 4 weeks	Cognition^a^ QoL Physical function

Abbreviations: *ACSM* American College of Sports Medicine, *BC* Body composition, *ECOG* Eastern Cooperative Oncology Group, *HRR* Heart rate reserve, *min.* Minutes, *No.* Number, *NR* Not reported, ^a^Primary outcome, *PA* Physical activity, *PC* Physical capacity, *PS* Performance status, *QoL* Quality of life, *Rep.* Repetitions, *RT* Resistance training, *SBT* Sedentary behavior time, ^b^Self-reported

^c^Participants were instructed to pedal a bicycle ergometer to match the target speed arbitrarily displayed on a computer screen. A standard path corresponding to the target number of revolutions was displayed on the computer screen (standard number of revolutions), and the participants pedaled the bicycle ergometer while visually tracking and paying attention to following the path

### Baseline characteristics

Table 2 details the baseline characteristics of the included studies. The studies involved a total of 412 participants with a mean age ranging from 68 to 77 years. Across all studies, breast (*N* = 159), prostate (*N* = 72) and colorectal cancer (*N* = 66) were the most frequent diagnoses. Sample sizes in the included studies ranged from 14 to 301 parti-cipants. The primary outcome was physical function in two studies [30, 32], and cognitive functioning in one study [33]. In these three studies, power calculations were presented [30, 32, 33]. Meanwhile, only one study achieved the desired power [33], while two did not due to higher number of drop-outs [30], standard deviations and baseline differences [32] than expected. In one study, neither the primary outcome of interest nor a power calculation were described [31].

**Table 2:** Demographic and medical characteristics of participants

Author, year	No. of participants	Age Mean (SD)	Sex Female/male	Diagnosis	Treatment setting and regimens	Performance status	Comorbidities
**Arrieta** et al [30], 2019	Total: 301 (300 analyzed) IG: 150 CG: 150	IG: 76.8 (5.1) CG: 76.6 (5.0)	180/120 60%/40%	Various cancers (colon, rectum, breast, esophagus, oral, kidney, prostate, bladder, lung, stomach, biliary ducts, ovary, hepato-cellular carcinoma, lymphoma, womb, endometrium, and pancreas) Cancer stage: NR	Curative treatments Systemic treatments: chemotherapy (60%), endocrine therapy (20%), targeted therapy (2%)	By ECOG PS 0: 53% PS 1: 30% PS 2: 4% NR: 13%	By CIRS-G: No grade 3–4 comorbidities: 79% 1 grade 3–4 comorbidity: 17% ≥2 grade 3–4 comorbidities: 4%
**Maréchal** et al [31], 2019	Total: 14 IG: 6 CG: 8	IG: 67.7 (2.1) CG: 69.6 (4.1)	11/3 79%/21%	Breast and colon cancer Cancer stage: NR	Systemic oncological treatment that started ≤12 weeks (treatment setting not further reported)	NR	NR
**Sajid** et al [32], 2016	Total: 19 EXCAP: 6 Wii: 8 CG: 5	EXCAP: 75.7 (9.5) Wii: 77.5 (6.7) CG: 71.8 (5.0)	0/19 0%/100%	Prostate cancer Cancer stage: NR	Treatment with ADT for ≥3 months Treatment setting: stable disease^a^	By Karnofsky, mean (SD): EXCAP: 88.3 (13.3) Wii: 87.5 (8.9) CG: 88 (16.4)	No. of comorbidities: EXCAP; 0–1: 67%, 2: 33% Wii; 0–1: 75%, 2: 25% CG; 0–1: 60%, 2: 40%
**Miki** et al [33], 2014	Total: 78 IG: 38 CG: 40	IG: 73.0 (4.6) CG: 75.5 (6.6)	43/35 55%/45%	Breast and prostate cancer Cancer stage: IG: Stage I: 28.9% Stage II: 57.9% Stage III: 2.6% Stage IV: 10.5% CG: Stage I: 32.5% Stage II: 37.5% Stage III: 12.5% Stage IV: 17.5%	Curative and palliative treatments Systemic treatment: endocrine therapy (53%), chemotherapy (6%)	NR	NR

Abbreviations: *ADT* Androgen deprivation therapy, *CG* Control group, *CIRS-G* Cumulative Illness Rating Scale-Geriatric, *ECOG* Eastern Cooperative Oncology Group, *IG* Intervention group, *No.* Number, *NR* Not reported, *PS* Performance status, *PSA* Prostate specific antigen, *SD* Standard deviation

^a^Stable or declining PSA, no progressive clinical symptoms, and no new metastatic lesions for one month prior to inclusion

In the study by Arrieta et al [30], all participants were curatively treated. Miki et al [33] included patients receiving both curative and palliative treatments. In both studies > 50% of the participants received systemic oncological treatments [30, 33]. Maréchal et al [31] included patients who received systemic oncological treatment that was started ≤12 weeks, with no further details provided regarding treatment setting. Finally, Sajid et al [32] included patients with prostate cancer who were treated with androgen deprivation therapy for ≥ 3 months and with stable disease.

### Exercise interventions

Interventions in the included studies were characterized by large differences. Two studies involved home-based and unsupervised interventions [30, 32], one was fully supervised with a personal instructor [33], and one was both supervised and unsupervised [31]. The intervention in the study by Arrieta et al [30] contained individualized phone advice on both resistance and aerobic exercises with the aim to maintain fitness. Advice on exercise frequency was given twice weekly, but hereafter all advice regarding exercise frequency, duration and intensity was individualized and not further described [30]. The study by Sajid et al [32] contained two intervention arms; the first arm, referred to as ‘EXCAP’, included a walking program and progressive resistance training with elastic bands. The walking program was of moderate intensity meeting 60–70% of heart rate reserve (HRR). All participants were instructed to increase their daily steps by 5–20% and to reach 10,000 steps per day. Pedometers and activity diaries were used for self-monitoring. The resistance training program was of low to moderate intensity. The starting point was tailored to each of the participants, but all participants were encouraged to progressively increase to a maximum of 4 sets of 15 repetitions. The second intervention arm, referred to as ‘Wii’, was designed to deliver a similar mode, intensity and duration as EXCAP, but was delivered with Wii-fit technology and also included balance exercises [32]. The supervised intervention from Miki et al [33] involved speed-feedback therapy where participants pedaled a bicycle ergometer and followed the speed and path that was visually shown on a display. The exercise load was set at 20 watts with a maximum of 80 rpm [33]. In the study conducted by Maréchal et al [31], the intervention consisted of both aerobic and resistance training [31]. A progression period of 4 weeks was given to attain 40 min of aerobic workout at 70–75% of HRR and was then maintained. The resistance training comprised five exercises targeting the major muscle groups. Two to three sets with 10–15 repetitions were performed at 50–65% of participant perceived maximum [31]. Duration of the interventions in the included studies varied from four weeks to twelve months with exercise frequencies ranging between once weekly to at least five times a week. Details about the interventions are summarized in Table 1.

Three studies compared the interventions with a control group (CG) receiving usual care [30, 32, 33], while Maréchal et al [31] used an active stretching group as control.

### Risk of bias

Overall, the studies applied proper randomization procedures. However, in the study by Maréchal et al [31] the randomization procedure was unclear, as participants were recruited from another trial. Blinding of participants were not applied in any of the studies [30–33] due to the natural difficulties of blinding participants in exercise-based trials. Blinding of outcome assessors was clearly documented in two studies [30, 33]. The trial context generates a risk of participants in the control groups performing exercises on their own, which could blur results of the interventions. Sajid et al [32] used diaries and pedometer assessments for all participants, which partly provided an overview of PA performed by all participants. Overall, the risk of bias was assessed as being “high” in one study (as four out of five domains were assessed as being of “some concerns”) [31], while the overall risk of bias assessment of three studies were “some concerns” [30, 32, 33]. The full risk of bias assessment is shown in Additional file 2: Appendix B.

### Effects

The study results on physical and cognitive outcomes are shown in Table 3.

**Table 3:** Study results at post intervention; physical and cognitive outcomes

Author, year	Functional tests		Strength tests		Physical activity		Cognition	
	Measure	Results	Measure	Results	Measure	Results	Measure	Results
**Arrieta** *et al* [30]*,* 2019	SPPB, loss of ≥ 1 SPPB point (%)	IG: 37.8% CG: 29.2% *p* = 0.772	-	-	IPAQ, median	IG: change from 2640 to 2133 CG: change from 2280 to 2772 *p* = 0.856	Verbal fluency, 15 sec., median (IQR)	IG: change from 6 (5-8) to 6 (5-8) CG: change from 6 (5-8) to 7 (6-8) *p* = 0.920
	SPPB score, median (IQR)	IG: change from 10 (8-11) to 10 (7.5-12) CG: change from 10 (8-11) to 10 (9-11) *p* = 0.999	-	-	-	-	Verbal fluency, 15-60 sec., median (IQR)	IG: change from 9 (7-11) to 9 (7-12) CG: change from 9 (6-11) to 9 (7-12) *p* = 0.945
	Gait speed (m/s), median	IG: change from 0.78 to 0.91 CG: change from 0.80 to 0.92 *p* = 0.171	-	-	-	-	-	-
**Maréchal** *et al* [31]*,* 2019	8-foot up & go (sec.), change, mean (SE)	IG: - 0.2 (0.3) CG: - 1.1 (0.5) *NS*	Chair stand test (rep.), change, mean (SE)	IG: 4.3 (0.9) CG: 1.0 (0.5) *p* = 0.01	SBT (min/week), change, mean (SE)	IG, change: - 295.7 (95.4) CG, change: - 11.3 (102.7) *NS*	-	-
	Sit & reach test (cm), change, mean (SE)	IG: 1.2 (1.6) CG: 0.8 (1.1) *NS*	Arm curl test (rep.), change, mean (SE)	IG: 2.7 (0.8) CG: 1.9 (0.7) *NS*	PASE score, change, mean (SE)	IG, change: 26.8 (34.7) CG, change: 19.2 (11.5) *NS*	-	-
	Six-minute walk test (m), change, mean (SE)	IG: 74.8 (19.5) CG: 63.8 (15.8) *NS*	Grip strength (kg), change, mean (SE)	IG: 5.8 (2.7) CG: - 0.6 (1.7) *NS*	-	-	-	-
	GPCS, change, mean (SE)	IG: 4.0 (0.2) CG: 1.5 (0.8) *p* = 0.047	Leg strength, 1RM, change, mean (SE)	IG: 8.8 (14.2) CG: 6.0 (5.9) *NS*	-	-	-	-
**Sajid** *et al* [32], 2016	SPPB, points, mean (SE)	EXCAP: change from 8.7 (0.9) to 10.0 (0.7) CG: change from 7.8 (1.1) to 8.4 (0.9) *p* = 0.403 Wii: change from 9.1 (0.6) to 9.1 (0.5) CG: change from 7.8 (1.1) to 8.4 (0.9) *p* = 0.400	Chest press reps, mean (SE)	EXCAP: change from 78.8 (9.0) to 82.9 (9.2) CG: change from 72.0 (6.1) to 73.5 (5.8) *p* = 0.727 Wii: change from 59.1 (3.6) to 63.8 (3.3) CG: change from 72.0 (6.1) to 73.5 (5.8) *p* = 0.459	Steps, mean (SE)	EXCAP: change from 3593.9 (864.5) to 5544.3 (1258.8) CG: change from 2625.7 (721.7) to 2242.3 (694.1) *p* = 0.019 Wii: change from 2999.9 (770.1) to 4223.7 (893.6) CG: change from 2625.7 (721.7) to 2242.3 (694.1) *p* = 0.051	-	-
	-	-	Handgrip strength, mean (SE)	EXCAP: change from 33.8 (3.4) to 35.5 (4.0) CG: change from 34.7 (2.2) to 34.2 (1.3) *p* = 0.447 Wii: change from 29.1 (1.8) to 30.7 (2.3) CG: change from 34.7 (2.2) to 34.2 (1.3) *p* = 0.285	-	-	-	-
**Miki** *et al* [33]*,* 2014	ADL; Barthel Index, mean (SE)	IG: change from 100.00 (0.00) to 100.00 (0.00) CG: change from 99.75 (0.25) to 99.75 (0.25) *p* = 0.333	-	-	-	-	Frontal Assessment Battery, mean (SE)	IG: change from 15.00 (0.26) to 16.61 (0.22) CG: change from 14.50 (0.30) to 14.95 (0.36) *p* = 0.003
	IADL; Lawton & Brody, mean (SE)	IG: change from 9.55 (0.18) to 9.74 (0.13) CG: change from 9.15 (0.24) to 9.28 (0.22) *p* = 0.097	-	-	-	-	-	-

*Abbreviations*: *ADL* activities of daily living, *CG* control group, *GPCS* Global Physical Capacity Score, *IADL* Instrumental Activities of Daily Living, *IG* intervention group, *IPAQ* International Physical Activity Questionnaire, *IQR* interquartile range, *m* meters, *m/s* meters per second, *min/week* minutes per week, *NS* no significant difference, *PASE* Physical Activity, *Rep.* repetitions, *RM* repetition maximum, *SE* standard error, *sec.* seconds, *SPPB* Short Physical Performance Battery, *SBT* sedentary behavior time

#### Health-related quality of life

Miki et al [33] measured the effect on HRQoL using the Functional Assessment of Cancer Therapy - General (FACT-G) and found no statistically significant differences between the groups.

#### Physical function

All studies reported results on physical functioning. Two studies used the Short Physical Performance Battery (SPPB) which is a group of measures combining results from gait speed, balance, and the chair stand test [30, 32]. In the study by Sajid et al [32], there were no statistically significant differences in SPPB between groups from baseline to post-intervention (6 weeks). However, a mixed effects linear regression model found a mean increase over time of 1.2 points in EXCAP, compared to a nearly constant score (exact numbers not reported) in the CG (*p* = 0.038), while there was no statistically significant difference in the change of scores between Wii and the CG. Arrieta et al [30] found no statistically significant differences in SPPB between groups after the 12-months intervention. However, after 24 months 40.3% of participants in the CG and 24.1% in the intervention group (IG) had declined ≥1 point in SPPB score, *p* = 0.057. In addition, in subgroup analysis they found a statistically significant difference in SPPB in favor of the IG in women with breast cancer (decline in SPPB; CG: 45.2% vs IG: 10.7%, *p* = 0.006) and in participants with a normal nutritional status according to the Mini Nutritional Assessment (decline in SPPB; CG: 50.0% vs IG: 19.4%, *p* = 0.009) [30].

Maréchal et al [31] used the Global Physical Capacity Score (GPCS) combining six tests for function, strength and aerobic capacity and comparing score sums with a reference population and found a statistically significant difference between groups; IG: 4.0 (SE 0.2) vs CG: 1.5 (SE 0.8), *p* = 0.047. Furthermore, they investigated effects on physical function using the six-minute walk test. While test results were improved in both the CG (mean increase: 63.8 m (m), SE 15.8) and in the IG (mean increase: 74.8 m, SE 19.5), there was no statistically significant difference between the groups [31]. On the remaining tests regarding physical function, no statistically significant differences between groups were found for Activities of Daily Living (ADL) [33], Instrumental Activities of Daily Living (IADL) [33], Sit & Reach test [31], or 8-ft up and go test [31].

#### Aerobic capacity

None of the included studies reported on aerobic capacity.

#### Muscle strength

Muscle strength was measured in two studies. Maréchal et al [31] demonstrated an improvement in the chair stand test of 4.3 repetitions (rep.) (SE 0.9) in the IG, compared to an improvement in the CG of 1.0 rep. (SE 0.5), *p* = 0.01. No statistically significant differences were found between groups in the arm curl test or for one repetition maximum (1RM) leg press [31]. Sajid et al [32] found no statistically significant differences between groups in grip strength or in chest press.

#### Body composition

One study [32] included body composition using dual energy x-ray absorptiometry (DEXA) scans and found no differences between groups in lean body mass.

#### Cancer and treatment-related symptoms and side effects

None of the included studies measured potential effects on symptoms and side effects.

#### Feasibility

Results regarding feasibility are shown in Table 4. The mean recruitment rate, as reported by two of the included trials [30, 33], was 74%. Patients were recruited through outpatient clinics at the hospitals [30, 32, 33], and from another exercise-based trial [31]. Barriers concerning recruitment were documented in one study; Miki et al [33] reported the following barriers: distance to hospital (31%), too busy (29%), poor physical condition (22%), not interested (10%) and other reasons (7%). Exercise adherence was reported by two studies; in the study by Arietta et al [30], the percentage of completed phone consultations was 81%, while the percentage of performed exercises was 70%. In the study by Miki et al [33], all participants completed all speed-feedback bicycle sessions. Three studies reported on adverse events [31–33]; all with no incidents of any events.

**Table 4:** Feasibility

Author, year	No. of patients eligible for the study	Included patients (% of eligible)	No. of patients completed post-test (%)	Statistical power calculation; estimated number of participants (actually included/planned)	Exercise adherence	Adverse events
**Arrieta** et al [30], 2019	368	301 (82%)	249 (83%)	Power calculation presented; (301/300)	Completed phone calls: 81.1% SR performance of PA: 70.1%	NR
**Maréchal** et al [31], 2019	(NR/secondary analysis)	14 (NR/secondary analysis)	14 (100%/ secondary analysis)	NR	NR	No adverse events
**Sajid** et al [32], 2016	NR	19 (NR)	18 (95%)	Power calculation presented; (19/18)	NR	No adverse events
**Miki** et al [33], 2014	146	78 (53%)	78 (100%)	Power calculation presented; (78/62 participants)	Feedback ergometer sessions: 100%	No adverse events

Abbreviations: *No.* Number, *NR* Not reported, *PA* Physical activity, *SR* Self-reported

The average attrition rate at the end of the intervention period, as reported by three studies [30, 32, 33], was 7%, while it on average was 35% in the full study period including follow-up [30, 32].

#### Clinical outcomes

Only one study investigated mortality and other clinical outcomes. Arietta et al [30] found no differences between groups regarding falls (IG: 7% vs CG: 7%), hospitalizations (IG: 18% vs CG: 16%), institutionalizations (IG: 9% vs CG: 4%) or mortality (IG: 10% vs CG: 11%).

#### Physical activity

Three studies investigated the effects on physical activity (PA). Sajid et al [32] found an increase of daily steps in EXCAP of 1950 steps, compared to a decrease of daily steps in the CG of 383 steps (*p* = 0.019), while there was no statistically significant difference between Wii (+ 1224 steps) and the CG (− 383 steps), *p* = 0.051. In the remaining two studies using self-reported PA, no statistically significant differences between groups were found [30, 31].

#### Cognition

Two studies measured effects on cognition. Miki et al [33] assessed cognitive function using the Frontal Assessment Battery. Participants in the IG had a mean change from 15.00 (SE: 0.26) to 16.61 (SE: 0.22), while the scores in the CG changed from 14.50 (SE: 0.30) to 14.95 (SE: 0.36), *p* = 0.003. In the study by Arrieta et al [30] no differences between groups were found for cognition measured by verbal fluency.

### Ongoing trials

The search on clinicaltrials.gov identified five studies, while an additional study was included as a published protocol article [34]. The inclusion age in three studies was ≥65 years, whereas three studies included patients ≥70 years. Two studies focused on patients with breast cancer, two studies included patients with hematologic malignancies, one study included patients with advanced pancreatic, biliary tract and lung cancer, and finally one study focused on patients with advanced lung and pancreatic cancer. All studies investigated the effect of a multimodal exercise program including aerobic and resistance training. The primary outcomes are lower body extremity strength (two studies), treatment tolerance/adherence (two studies), joint pain (one study) and disability-free survival (one study). Sample sizes ranged from 76 to 130 participants. The search was last updated on December 9, 2019. An overview of the studies registered at clinicaltrials.gov is shown in Table 5.

**Table 5:** Ongoing RCTs from clinicaltrials.gov investigating the effects of exercise-based interventions exclusively in older patients (≥ 65 years) with cancer undergoing medical antineoplastic treatment

Trial identifier	Title	Investigator, country	Cancer diagnoses	Treatment setting	No.	Age	Exercise type and duration	Duration	Delivery of intervention	Primary outcome	Study status
NCT03656731	Exercise in older women with breast cancer during systemic therapy – BREACE	Lund, Denmark	Breast cancer	(Neo)adjuvant or first or second-line palliative systemic treatment	100	≥ 65 years	Team-based progressive resistance training + homed-based walking	12 weeks	Supervised and unsupervised	Lower body extremity strength	Recruiting Study completion: December 2021
NCT03411200	Patient activation through counseling, exercise and mobilization (PACE-Mobil)	Mikkelsen, Denmark	Pancreatic, biliary tract, and lung cancer	Palliative treatment with CT, IT or TT. ≤ 3 months of diagnosis	100	≥ 65 years	Team-based progressive resistance training + homed-based walking	12 weeks	Supervised and unsupervised	Lower body extremity strength	Recruiting Study completion: January 2021
NCT03955627	REJOIN trial for older breast cancer survivors	Bluethmann, USA	Stage I, II or III breast cancer	Completed surgery, radiation and/or CT. Planning to initiate treatment with aromatase inhibitors	76	≥ 65 years	Exercise consultation and the Fit & Strong exercise program; low-impact aerobic exercise and resistance training. Bi-weekly exercise (60 min.) and educational sessions (30 min.)	16 weeks	Supervised (8 weeks) and unsupervised (8 weeks)	Change in pain (joint pain)	Not yet recruiting Study completion: January 2022
NCT04057443	Nutritional and physical exercise intervention in older patients with malignant hemopathies (ICOSENIORHEM)	Antonio, Spain	Myelodysplastic syndromes, lymphoproliferative syndromes, and multiple myeloma	Recently diagnosed	80	≥ 70 years	Group-based program of “mixed structure”. + nutritional support	During treatment or max. 6 months.	Supervised	Adherence to oncological treatment	Recruiting Study completion: June 2021
NCT03100175	Strength and aerobic training in elderly lymphoma patients during chemotherapy and its impact on treatment outcomes, patients functioning and biological markers of aging	Vaxman, Israel	Large B cell lymphoma, Hodgkin lymphoma, or follicular lymphoma	Newly diagnosed. Planned for at least 75% of full dose CT	100	≥ 70 years	Homed-based resistance training, brisk walking, and balance exercises + telephone call 2 x weekly	NR	Unsupervised	Treatment tolerance	Unknown status Last registered study completion date: March 2019
Study protocol published in BMC cancer [31] PMID: 31151425	A randomized phase II study of nutritional and exercise treatment for elderly patients with advanced non-small cell lung or pancreatic cancer: the NEXTAC-TWO study	Miura, Japan	Advanced non-small cell lung cancer or inoperable pancreatic cancer	Scheduled for systemic cancer therapy (i.e. CT, and/or TT and/or IT	130	≥ 70 years	Muscle training (3–5 exercises, daily training), physical activity advice, and nutritional counseling and supplements, which are all individualized.	12 weeks + follow-up (including advice) until death or disability	Unsupervised	Disability-free survival	Recruitment ongoing at February 26, 2019

Abbreviations: *CT* Chemotherapy, *IT* Immunotherapy, *min.* Minutes, *No.* Number, *NR* Not reported, *RCTs* Randomized controlled trials, *TT* Targeted therapy

## Discussion

In this systematic review, we found some evidence for beneficial effects from exercise interventions on physical function, muscle strength, physical activity and cognitive function among older patients with cancer during antineoplastic treatment. We found no evidence for any effects of exercise on HRQoL, aerobic capacity, body composition, cancer-related symptoms and side effects, or for any clinical outcomes. Overall, the results were inconclusive due to lack of sufficiently powered studies.

Patients with cancer are recommended to avoid inactivity and to return to normal activities as soon as possible following diagnosis [35]. Though it is often stated that patients with cancer can perform exercise according to general recommendations for healthy people, published guidelines also emphasize the current knowledge gaps, here among lack of evidence regarding safety and effects of exercise for older patients with cancer [16, 35]. Despite recommendations of staying physically active after a cancer diagnosis, most patients with cancer and cancer survivors decrease PA level and struggle to meet PA recommendations [36, 37]. In a longitudinal study conducted by De Groef et al [38], self-reported levels of activity were investigated among 267 patients with breast cancer before surgery and at several following time points. After 2 years, all activity levels (total, sports, occupational and household) were statistically significantly lower compared to preoperative levels [38].

In a qualitative study focusing on exploring attitudes towards PA among older patients (≥ 65 years) with cancer during systemic oncological treatments, several barriers towards PA and exercise were identified, including physical limitations due to age-related declines in health, fatigue, and comorbidities [39]. Thus, recruitment to and adherence in exercise-based studies focusing on older patients with cancer may be challenging.

In the current review, rates of recruitment were only reported by two studies and ranging from 53% [33] to 82% [30]. Description of reasons for excluding patients in the screening process and reasons why patients declined, was not described in three studies. Knowledge about these factors are important and could guide future trials. Only two studies reported on adherence to exercise sessions, with Miki et al [33] reporting 100% adherence, and Arrieta et al [30] reported 70% adherence by self-report. Estimates of PA based on self-report are generally higher than estimates derived from objective measures [40, 41]. Therefore, self-reported adherence must be interpreted with caution.

Even though some statistically significant effects were found in individual studies regarding functional capacity and lower extremity muscle strength [31, 32], increased activity level [32], and cognitive improvement [33], no evidence for an effect was seen for most outcomes, and therefore the overall evidence of positive effects were sparse. There could be several explanations for this. First, due to the small sample sizes in some of the studies, there could be a risk of type II error due to lack of statistical power. Second, non-adherence to the intervention or contamination by increased activity among participants in the control groups could have negated differences between groups. Exercise-based trials may be particularly susceptible to contamination, as patients who accept participation presumably are motivated to exercise, and therefore typically unblinded. Due to lack of reporting on adherence in two studies [31, 32] and the uncertainty regarding control participants’ physical activities, it was not possible to determine if non-adherence and/or contamination could explain the lack of documented effects in most outcomes.

Another explanation for the lack of effect could be that the duration and/or intensity of the exercise programs were too short/low to make statistically significant differences. Although the intervention period in Arietta et al was long (12 months), the exercise intensity was unknown as it was adjusted to each participant’s motivation and capability and was not further described [30]. In the study by Maréchal et al [31], the prescribed exercise intensities were well described. However, no reports on actual exercise adherence was provided [31]. Miki et al [33] investigated the effect of four speed-feedback sessions. Even though the intervention was targeted at improving cognitive function, the study was included in this systematic review as it involved some degree of exercising. However, with only four sessions, each with a pedaling time of 5 min and an exercise load at 20 watts, the intervention is unlikely to have any effects on physical outcomes. The secondary outcomes included in the study (ADL, IADL and FACT-G) could potentially change due to cognitive improvements; yet no statistically significant differences were found, possibly due to the overall good functional status of the participants [33]. Sajid et al [32] provided detailed descriptions of the exercise intensities regarding both the walking program and the resistance exercises. However, while participants in the EXCAP group statistically significantly increased their number of daily steps, no other reports on exercise adherence were provided [32].

Two of the included trials were home-based [30, 32]. While research has shown that most patients with cancer or cancer survivors prefer home-based exercise [42], it has also been demonstrated that supervised exercise programs are more effective than non-supervised [43]. In a systematic review investigating exercise preferences among older adults, preferences to exercise settings and social contexts varied [44]. However, accessibility of the location seemed to be more important than the type of location [44]. Hence, accessibility should be carefully considered in future exercise-based trials among older patients with cancer.

Overall, the included studies used relevant outcome variables and assessment methods. However, only one study used patient reported outcome measures (PROMs) to investigate the effect on HRQoL; Miki et al [33] measured the effect on FACT-G and found no statistically significant differences between groups. The use of PROMs in health care research has increased in the recent years [45]. By using PROMs, researchers can capture outcomes that are highly important to patients, such as physical and psychological health, social functioning, and distress from symptoms and side effects [45]. Therefore, it must be considered as highly relevant to include PROMs in future exercise-based intervention studies among older patients with cancer.

### Strengths and limitations

This systematic review has some limitations. The large differences between studies in patient characteristics, interventions, outcome variables and assessment methods did not support the use of meta-analysis. Therefore, in accordance with the Cochrane Handbook [46], the results were described narratively. The inclusion of the speed-feedback study conducted by Miki et al [33] is contentious due to the very limited physical exercise component in the intervention. However, as it fulfilled all inclusion criteria, the study was included. Nevertheless, the results from this study must be interpreted with caution as it primarily investigates cognitive training. In hindsight, a more precise definition of the eligibility criteria and exercise interventions would have been preferable.

Even though the studies in this review solely included patients with cancer ≥65 years, it must be emphasized that most participants had few comorbidities, good PS, and no cognitive deficits. Therefore, the results may not be generalizable to all patients in the older cancer population.

In two of the included studies more than 50%, but not all participants, received systemic oncological treatment [30, 33]. Therefore, the results from these two trials must be interpreted with caution in answering our research question.

We only searched for and included RCTs that solely focused on patients ≥65 years, which was limited to four studies. In addition, two studies had very small sample sizes (< 20), which increases the risk of biased results. In hindsight, several exercise-based RCTs have been conducted among patients with cancer, and even if older patients in general are underrepresented in exercise-based trials, some trials have a reasonable representation of older individuals [47, 48] and/or may provide age-divided results.

Strengths of the review include the systematic approach guided by the Cochrane Handbook. The search was conducted in four databases, and the included studies were reviewed and assessed by two authors firstly independent and then by discussion to consensus.

### Implications

Larger RCTs of high-quality are needed to further investigate the effect of exercise training on physical function, physical capacity, and HRQoL among older patients with cancer during systemic oncological treatment.

## Conclusion

We have summarized the effects of exercise on HRQoL, physical function, aerobic capacity, and additional physical, cognitive and clinical outcomes among older patients (≥ 65 years) with cancer during systemic oncological treatment. Evidence for the effect of exercise interventions in this population is limited. Reasons for the limited effects could be caused by failing intervention, but also reflect limitations in the included studies, including small sample sizes. In all, the effects of exercise in older patients with cancer receiving medical antineoplastic treatments are inconclusive.

## Supplementary information

**Additional file 1 Appendix A.** Applied words in the systematic literature search.

**Additional file 2 Appendix B.** Risk of Bias Assessment.

## Data Availability

All data generated or analyzed during this study are included in this published article and its supplementary files.

## Abbreviations

ADL: Activities of Daily Living
CG: Control group
DEXA: Dual energy x-ray absorptiometry
ECOG: Eastern Cooperative Oncology Group
FACT-G: Functional Assessment of Cancer Therapy – General
GPCS: Global Physical Capacity Score
GRADE: The Grading of Recommendation, Assessment, Development and Evaluation tool
HRQoL: Health-Related Quality of Life
HRR: Heart rate reserve
IADL: Instrumental Activities of Daily Living
IG: Intervention group
IQR: Interquartile range
m: Meters
PA: Physical activity
PRISMA: Preferred Reporting Items for Systematic Reviews and Meta-Analysis
PROMs: Patient-reported outcome measures
PS: Performance status
RCT: Randomized controlled trial
Rep.: Repetitions
rpm: Revolutions per minute
SE: Standard error
SPPB: Short Physical Performance Battery
VO2max: Maximal oxygen uptake
1RM: One repetition maximum

## References

[1] McTiernan A, Friedenreich CM, Katzmarzyk PT, Powell KE, Macko R, Buchner D (2019). Physical activity in cancer prevention and survival: a systematic review. Med Sci Sports Exerc.

[2] Schmid D, Behrens G, Keimling M, Jochem C, Ricci C, Leitzmann M (2015). A systematic review and meta-analysis of physical activity and endometrial cancer risk. Eur J Epidemiol.

[3] Boyle T, Keegel T, Bull F, Heyworth J, Fritschi L (2012). Physical activity and risks of proximal and distal colon cancers: a systematic review and meta-analysis. J Natl Cancer Inst.

[4] Psaltopoulou T, Ntanasis-Stathopoulos I, Tzanninis IG, Kantzanou M, Georgiadou D, Sergentanis TN (2016). Physical activity and gastric cancer risk: a systematic review and meta-analysis. Clin J Sport Med.

[5] Al-Bayati O, Hasan A, Pruthi D, Kaushik D, Liss MA (2019). Systematic review of modifiable risk factors for kidney cancer. Urol Oncol.

[6] Al-Zalabani AH, Stewart KF, Wesselius A, Schols AM, Zeegers MP (2016). Modifiable risk factors for the prevention of bladder cancer: a systematic review of meta-analyses. Eur J Epidemiol.

[7] Chen X, Wang Q, Zhang Y, Xie Q, Tan X (2019). Physical activity and risk of breast cancer: a meta-analysis of 38 cohort studies in 45 study reports. Value Health.

[8] Singh S, Devanna S, Edakkanambeth Varayil J, Murad MH, Iyer PG (2014). Physical activity is associated with reduced risk of esophageal cancer, particularly esophageal adenocarcinoma: a systematic review and meta-analysis. BMC Gastroenterol.

[9] Wu W, Guo F, Ye J, Li Y, Shi D, Fang D (2016). Pre- and post-diagnosis physical activity is associated with survival benefits of colorectal cancer patients: a systematic review and meta-analysis. Oncotarget..

[10] Ballard-Barbash R, Friedenreich CM, Courneya KS, Siddiqi SM, McTiernan A, Alfano CM (2012). Physical activity, biomarkers, and disease outcomes in cancer survivors: a systematic review. J Natl Cancer Inst.

[11] Schmid D, Leitzmann MF (2014). Association between physical activity and mortality among breast cancer and colorectal cancer survivors: a systematic review and meta-analysis. Ann Oncol.

[12] Buffart LM, Kalter J, Sweegers MG, Courneya KS, Newton RU, Aaronson NK (2017). Effects and moderators of exercise on quality of life and physical function in patients with cancer: an individual patient data meta-analysis of 34 RCTs. Cancer Treat Rev.

[13] Gerritsen JK, Vincent AJ (2016). Exercise improves quality of life in patients with cancer: a systematic review and meta-analysis of randomised controlled trials. Br J Sports Med.

[14] Klepin HD, Mohile SG, Mihalko S (2013). Exercise for older cancer patients: feasible and helpful?. Interdiscip Top Gerontol.

[15] Grimmett C, Corbett T, Brunet J, Shepherd J, Pinto BM, May CR (2019). Systematic review and meta-analysis of maintenance of physical activity behaviour change in cancer survivors. Int J Behav Nutr Phys Act.

[16] Kilari D, Soto-Perez-de-Celis E, Mohile SG, Alibhai SM, Presley CJ, Wildes TM (2016). Designing exercise clinical trials for older adults with cancer: recommendations from 2015 cancer and aging research group NCI U13 meeting. J Geriatr Oncol.

[17] Forbes CC, Swan F, Greenley SL, Lind M, Johnson MJ (2020). Physical activity and nutrition interventions for older adults with cancer: a systematic review. J Cancer Surviv.

[18] Pilleron S, Sarfati D, Janssen-Heijnen M, Vignat J, Ferlay J, Bray F (2019). Global cancer incidence in older adults, 2012 and 2035: a population-based study. Int J Cancer.

[19] Weinstein JR, Anderson S (2010). The aging kidney: physiological changes. Adv Chronic Kidney Dis.

[20] Bhutto A, Morley JE (2008). The clinical significance of gastrointestinal changes with aging. Curr Opin Clin Nutr Metab Care.

[21] Sharma G, Goodwin J (2006). Effect of aging on respiratory system physiology and immunology. Clin Interv Aging.

[22] Janssen I, Heymsfield SB, Wang ZM, Ross R (2000). Skeletal muscle mass and distribution in 468 men and women aged 18-88 yr. J Appl Physiol.

[23] Demontiero O, Vidal C, Duque G (2012). Aging and bone loss: new insights for the clinician. Ther Adv Musculoskelet Dis.

[24] Fried LP, Ferrucci L, Darer J, Williamson JD, Anderson G (2004). Untangling the concepts of disability, frailty, and comorbidity: implications for improved targeting and care. J Gerontol A Biol Sci Med Sci.

[25] Cheville AL, Mustian K, Winters-Stone K, Zucker DS, Gamble GL, Alfano CM (2017). Cancer rehabilitation: an overview of current need, delivery models, and levels of care. Phys Med Rehabil Clin N Am.

[26] Sterne JAC, Savovic J, Page MJ, Elbers RG, Blencowe NS, Boutron I (2019). RoB 2: a revised tool for assessing risk of bias in randomised trials. BMJ..

[27] Schünemann H, Brożek J, Guyatt G, Oxman A (2013). GRADE handbook. Introduction to GRADE handbook. Handbook for grading the quality of evidence and the strength of recommendations using the GRADE approach. Updated October 2013.

[28] Demark-Wahnefried W, Clipp EC, Morey MC, Pieper CF, Sloane R, Snyder DC (2006). Lifestyle intervention development study to improve physical function in older adults with cancer: outcomes from project LEAD. J Clin Oncol.

[29] Morey MC, Snyder DC, Sloane R, Cohen HJ, Peterson B, Hartman TJ (2009). Effects of home-based diet and exercise on functional outcomes among older, overweight long-term cancer survivors: RENEW: a randomized controlled trial. JAMA..

[30] Arrieta H, Astrugue C, Regueme S, Durrieu J, Maillard A, Rieger A (2019). Effects of a physical activity programme to prevent physical performance decline in onco-geriatric patients: a randomized multicentre trial. J Cachexia Sarcopenia Muscle.

[31] Marechal R, Fontvieille A, Parent-Roberge H, Fulop T, Riesco E, Pavic M (2019). Effect of a mixed-exercise program on physical capacity and sedentary behavior in older adults during cancer treatments. Aging Clin Exp Res.

[32] Sajid S, Dale W, Mustian K, Kotwal A, Heckler C, Porto M (2016). Novel physical activity interventions for older patients with prostate cancer on hormone therapy: a pilot randomized study. J Geriatr Oncol.

[33] Miki E, Kataoka T, Okamura H (2014). Feasibility and efficacy of speed-feedback therapy with a bicycle ergometer on cognitive function in elderly cancer patients in Japan. Psycho-oncology..

[34] Miura S, Naito T, Mitsunaga S, Omae K, Mori K, Inano T (2019). A randomized phase II study of nutritional and exercise treatment for elderly patients with advanced non-small cell lung or pancreatic cancer: the NEXTAC-TWO study protocol. BMC Cancer.

[35] Campbell KL, Winters-Stone KM, Wiskemann J, May AM, Schwartz AL, Courneya KS (2019). Exercise guidelines for cancer survivors: consensus statement from international multidisciplinary roundtable. Med Sci Sports Exerc.

[36] Craike M, Hose K, Livingston PM (2013). Physical activity participation and barriers for people with multiple myeloma. Support Care Cancer.

[37] Chou YJ, Lai YH, Lin BR, Liang JT, Shun SC (2017). Factors influencing amount of weekly exercise time in colorectal cancer survivors. Cancer Nurs.

[38] De Groef A, Geraerts I, Demeyer H, Van der Gucht E, Dams L, de Kinkelder C (2018). Physical activity levels after treatment for breast cancer: two-year follow-up. Breast..

[39] Mikkelsen MK, Nielsen DL, Vinther A, Lund CM, Jarden M (2019). Attitudes towards physical activity and exercise in older patients with advanced cancer during oncological treatment - a qualitative interview study. Eur J Oncol Nurs.

[40] Garriguet D, Tremblay S, Colley RC (2015). Comparison of physical activity adult questionnaire results with accelerometer data. Health Rep.

[41] Fukuoka Y, Haskell W, Vittinghoff E (2016). New insights into discrepancies between self-reported and accelerometer-measured moderate to vigorous physical activity among women - the mPED trial. BMC Public Health.

[42] Wong JN, McAuley E, Trinh L (2018). Physical activity programming and counseling preferences among cancer survivors: a systematic review. Int J Behav Nutr Phys Act.

[43] Sweegers MG, Altenburg TM, Chinapaw MJ, Kalter J, Verdonck-de Leeuw IM, Courneya KS (2018). Which exercise prescriptions improve quality of life and physical function in patients with cancer during and following treatment? A systematic review and meta-analysis of randomised controlled trials. Br J Sports Med.

[44] Amireault S, Baier JM, Spencer JR. Physical activity preferences among older adults: a systematic review. J Aging Phys Act. 2018:1–12. 10.1123/japa.2017-0234. Online ahead of print. 10.1123/japa.2017-0234 29283793

[45] Deshpande PR, Rajan S, Sudeepthi BL, Abdul Nazir CP (2011). Patient-reported outcomes: a new era in clinical research. Perspect Clin Res.

[46] Cumpston M, Li T, Page MJ, Chandler J, Welch VA, Higgins JP (2019). Updated guidance for trusted systematic reviews: a new edition of the Cochrane handbook for systematic reviews of interventions. Cochrane Database Syst Rev.

[47] Bjerre ED, Petersen TH, Jørgensen AB, Johansen C, Krustrup P, Langdahl B (2019). Community-based football in men with prostate cancer: 1-year follow-up on a pragmatic, multicentre randomised controlled trial. PLoS Med.

[48] Taaffe DR, Newton RU, Spry N, Joseph D, Chambers SK, Gardiner RA (2017). Effects of different exercise modalities on fatigue in prostate cancer patients undergoing androgen deprivation therapy: a year-long randomised controlled trial. Eur Urol.

