# Supplementary material for: The effect of exercise-based interventions on health-related quality of life and physical function in older patients with cancer receiving medical antineoplastic treatments: a systematic review
Source: Eur Rev Aging Phys Act. 2020 Oct 19;17:18. doi: 10.1186/s11556-020-00250-w (PMC7574419; doi:10.1186/s11556-020-00250-w)
Supplement: Supplementary file 1 — Additional file 1 Appendix A. Applied words in the systematic literature search. [file 11556_2020_250_MOESM1_ESM.docx]

**Appendix A. Applied words in the systematic literature search**

| Applied search words according to search focus a-d | | | |
| --- | --- | --- | --- |
| A: Exercise | B: Cancer | C: Older adults | D: Randomized controlled trials |
| Cardiovascular training [ti/ab]  Aerobic exercise* [ti/ab]  Sport* [ti/ab]  Sports [Mesh])  Crossfit [ti/ab]  Danc* [ti/ab]  Endurance training [ti/ab]  Exercise movement technique* [ti/Ab] Exercise Movement Techniques [Mesh] Exercise* [ti/ab]  Exercise [Mesh]  Exercise therap* [ti/ab]  Exercise Therapy [Mesh]  Physical fitness [ti/ab]  Jogging [ti/ab]  Jogg* [ti/ab]  Kinesiotherap* [ti/ab]  Martial art* [ti/ab]  Muscle strengthening [ti/ab]  Muscle training [ti/ab]  Strength training [ti/ab]  Resistance training [ti/ab]  Weight lifting [ti/ab]  Physical activit* [ti/ab]  Pilates [ti/ab]  Yoga [ti/ab]  Running [ti/ab]  Run* [ti/ab]  Step activit* [ti/ab]  Tai chi [ti/ab]  Tai ji [ti/ab]  Walking [ti/ab]  Walk* [ti/ab]  Water exercise* [ti/ab]  Water-based exercise* [ti/ab]  High-intensity interval training [ti/ab] Physical Therapy Modalities [Mesh] | Cancer* [ti/ab] Neoplasm* [ti/ab] Neoplasms [Mesh] | Aged [Mesh]  Aged, 80 and over [Mesh]  Geriatrics [Mesh]  Geriatric* [ti/ab]  Elder* [ti/ab]  Old age* [ti/ab]  Old-age* [ti/ab]  Aging [ti/ab]  Aged* [ti/ab]  Senior* [ti/ab]  Old* [ti/ab] | *Cochrane Collaboration highly sensitive search strategy* |

Abbreviations: ab; abstract, Mesh; Medical Subject Heading, ti; title
