# Supplementary material for: The effect of exercise-based interventions on health-related quality of life and physical function in older patients with cancer receiving medical antineoplastic treatments: a systematic review
Source: Eur Rev Aging Phys Act. 2020 Oct 19;17:18. doi: 10.1186/s11556-020-00250-w (PMC7574419; doi:10.1186/s11556-020-00250-w)
Supplement: Supplementary file 2 — Additional file 2 Appendix B. Risk of Bias Assessment. [file 11556_2020_250_MOESM2_ESM.docx]

**Appendix B. Risk of Bias Assessment**

| *Risk of bias assessed according to the following domains** | **Assessed by** | **Randomization process** | **Deviations from intended interventions** | **Missing outcome data** | **Measurement of the outcome** (primary outcome) | **Selection of the reported results** | **Overall risk of bias** |
| --- | --- | --- | --- | --- | --- | --- | --- |
| **Arietta** *et al* (30) | Article  Protocol article  Clinicaltrials.gov | Low | Some concerns | Low | Low | Low | **Some concerns** |
| **Maréchal** *et al* (31) | Article | Some concerns | Some concerns | Low | Some concerns | Some concerns | **High risk** |
| **Sajid** *et al* (32) | Article | Low | Low | Low | Some concerns | Some concerns | **Some concerns** |
| **Miki** *et al* (33) | Article | Low | Some concerns | Low | Low | Some concerns | **Some concerns** |

*Risk of bias was assessed in all studies and relevant pre-registrations according to the Revised Cochrane Risk-of-Bias Tool for Randomized Trials (RoB 2)
